# Diagnostic efficacy and necessity of 18F-FDG PET/CT in fever of unknown origin: insights from a retrospective cohort study

**DOI:** 10.3389/fmed.2024.1511710

**Published:** 2025-02-10

**Authors:** Xiaoman Yu, Shuang Wang, Na Du, Hongguang Zhao, Haiying Chen

**Affiliations:** ^1^Department of Infectious Diseases, The First Affiliated Hospital of Jilin University, Changchun, China; ^2^Department of Nuclear Medicine, The First Hospital of Jilin University, Changchun, China

**Keywords:** fever of unknown origin (FUO), 18F-FDG PET/CT, diagnostic value, image analysis, final diagnosis

## Abstract

**Background:**

Despite advancements in medical examination equipment and techniques, fever of unknown origin (FUO) remains challenging in internal medicine.

**Purpose:**

This study evaluates the diagnostic efficacy and necessity of 18F-fluorodeoxyglucose positron emission tomography/computed tomography (18F-FDG PET/CT) in patients with FUO.

**Methods:**

We retrospectively analyzed the results of 18F-FDG PET/CT in a cohort of 284 patients with FUO admitted to the Department of Infection at the First Hospital of Jilin University between January 2018 and March 2024. All patients received a final clinical diagnosis after various treatments, which helped determine the diagnostic relevance of identified lesions using 18F-FDG PET/CT. Additionally, univariate and multivariate logistic regression analyses were performed to evaluate the predictive value of relevant laboratory indices on the true-positive results of 18F-FDG PET/CT. The diagnostic performance for different etiologies of FUO was assessed by calculating the area under the receiver operating characteristic curve.

**Results:**

Of the 284 enrolled patients, infectious diseases were diagnosed in 53 (18.7%), non-infectious inflammatory diseases in 76 (26.8%), malignant tumors in 66 (23.2%), and 89 (31.3%) remained undiagnosed. The final diagnoses of 136 patients (47.9%) correlated with their 18F-FDG PET/CT results, yielding a sensitivity of 79.5%, specificity of 61.1%, positive predictive value of 75.6%, and negative predictive value of 66.3%. Furthermore, a correlation was found between localized pain, prolonged activated partial thromboplastin time, and true-positive 18F-FDG PET/CT results.

**Conclusion:**

The high diagnostic efficacy of 18F-FDG PET/CT in FUO suggests its potential as a routine imaging modality, which could enhance patient management and reduce the need for costly and unnecessary invasive procedures. The identification of clinical factors that are predictive of true-positive diagnosis could facilitate more effective allocation of PET/CT imaging.

## 1 Introduction

Fever of unknown origin (FUO) was first defined in 1961 by Petersdorf and Beeson as a recurrent fever exceeding 38.3°C, persisting for more than 3 weeks, and remaining undiagnosed after at least 1 week of hospitalization” (1). In 1991, Durack and Street revised these criteria to “include recurrent fevers above 38.3°C lasting over 3 weeks, which remain undiagnosed following either a three-day inpatient stay or 3 outpatient visits” (2). Currently, FUO is defined by (1) at least two episodes of fever ≥38.3°C (≥ 101°F), (2) an illness duration of ≥ 3 weeks, or multiple fever episodes within this timeframe; and (3) the absence of any known immunocompromised state (excluding patients with nosocomial infections, known HIV infections, or other immune-compromised conditions); (4) despite comprehensive history taking, physical examinations, and relevant testing, the diagnosis remains elusive (3).

Currently, more than 200 causes of FUO (50) are identified. Differential diagnosis traditionally categorizes these into four types: infectious diseases, non-infectious inflammatory diseases (NIID), malignant tumors, and miscellaneous causes. With advances in examination techniques, the precision of instruments, and broader scientific knowledge, the proportion of FUO cases attributed to infections and malignant tumors has decreased. Nonetheless, the rate of undiagnosed FUO cases in various studies still ranges from 7 to 53% (4).

FUO continues to pose significant challenges in internal medicine, often due to undetectable molecular, cellular, or microbial abnormalities in a patient’s blood or body fluids. While traditional non-invasive imaging tests such as X-rays, ultrasound, computed tomography (CT), and magnetic resonance imaging (MRI) can identify localized lesions, they may fall short in detecting early-stage diseases that exhibit primarily metabolic rather than anatomical changes. Therefore, these conventional imaging methods frequently lack accuracy in early-stage infections, inflammatory conditions, or patients with unaltered anatomy due to the disease process (5). Contrarily, 18F-FDG, a glucose analog, behaves similarly to glucose in the bloodstream and tissues, entering cells via GLU-1 to GLU-5 transporter proteins on the cell membrane. Once inside the cell, phosphorylated 18F-FDG cannot be further metabolized and thus remains trapped. Crucially, 18F-FDG uptake is not exclusive to tumor cells; it also occurs in all activated leukocytes (granulocytes, lymphocytes, and monocytes), which enables the imaging of both acute and chronic inflammatory processes (6). 18F-FDG PET can detect disease activity at the cellular and even molecular levels before morphological changes occur, and it can differentiate between active and inactive disease states, as well as distinguish between infections and sterile inflammatory or malignant tumors (7).

Following the introduction of PET/CT, integrating metabolic pathophysiological data with anatomical-pathological information from CT has significantly enhanced clinical disease diagnosis, improved anatomical resolution, and increased the accuracy of 18F-FDG PET (8). In clinical practice, 18F-FDG PET/CT is instrumental in diagnosing patients with FUO, offering high accuracy, sensitivity, resolution, and a brief interval between injection and imaging time (3, 9, 10).

For patients with FUO, enduring fever without identifying a specific cause imposes a significant economic burden and psychological strain. Therefore, timely diagnosis and appropriate treatment are crucial for improving the prognosis of patients with FUO.

## 2 Materials and methods

### 2.1 Patient population

In this study, we conducted a comprehensive search of the clinical database at the First Affiliated Hospital of Jilin University for patients with FUO who were admitted to the Department of Infectious Diseases and underwent 18F-FDG PET/CT scans between January 2018 and March 2024. All included patients met the current criteria for FUO, and despite exhaustive investigations—including detailed history taking, physical examination, and relevant laboratory testing—the cause of the fever remained undetermined. The laboratory tests conducted included routine blood and urine analysis, culture of various body fluids, measurement of ultrasensitive C-reactive protein and calcitoninogen levels, erythrocyte count determination, testing for EBV and cytomegalovirus antibodies or nucleic acids, rubella virus antibody and toxoplasmosis antibody testing, brucella agglutination tests, HIV tests, testing for *Mycobacterium tuberculosis* and non-tuberculosis mycobacteria, tuberculosis T-spot testing, and parasitic worm antibody and egg testing. Additional diagnostics included anti-nuclear and anti-neutrophil antibody screening, cyclic citrullinated peptide antibody testing, anti-cardiac phospholipid antibody screening, bone marrow smears, immunohistochemical examination, and imaging tests such as X-ray, ultrasound, CT, and MRI.

Ultimately, 284 patients were enrolled. We retrospectively analyzed the 18F-FDG PET/CT findings and clinicopathological data of these patients. All patients were followed for at least 3 months to establish a definitive diagnosis. Consistent with prior studies and consensus, symptoms of the disease typically manifest within this timeframe; hence, only diagnoses made within 3 months (11) post-18F-FDG PET/CT were deemed pertinent to the findings. The Regional Ethics Committee of the First Hospital of Jilin University approved this study (2024-1133). Given its retrospective nature, informed consent from the patients was deemed unnecessary.

### 2.2 18F-FDG PET/CT imaging

Patients were instructed to adhere to a high-fat, low-carbohydrate diet and fast for a minimum of 6 h before undergoing 18F-FDG PET/CT, ensuring glucose levels were below 11.1 mmol/L before administering the radiotracer. For those with cardiovascular implantable electronic devices or prosthetic heart valves, where cardiac infection is suspected to cause FUO, a specific preparatory regimen was initiated to inhibit glucose metabolism in cardiomyocytes. This involved starting a high-fat, low-carbohydrate diet 3 days before imaging (4).

An intravenous injection of 3.7 MBq/KG of 18F-FDG was administered 60 min before scanning, using an integrated PET/CT scanner (Siemens Biograph 16 HR, 2 min/bed). The scanning protocol covered from head to mid-thigh, with additional lower extremity scans performed as clinically necessary.

### 2.3 18F-FDG PET/CT image analysis

All 18F-FDG PET/CT images were reviewed by at least one experienced nuclear medicine physician and one radiologist, both familiar with the clinical data. A positive result was defined as 18F-FDG uptake intensity exceeding the physiologic biodistribution of the radiopharmaceutical in any anatomical structure not attributable to physiologic processes. Negative results indicated 18F-FDG uptake of only physiologic significance, with no pathological findings on CT images. Scans deemed inconclusive were reassessed by another nuclear medicine physician blind to the original image interpretation, the patient’s clinical history, and all associated laboratory and microbiological data.

### 2.4 Diagnostic reference criteria

The final diagnosis of a patient with fever of FUO relies not solely on the results of 18F-FDG PET/CT but on an integrative analysis encompassing various laboratory tests conducted during hospitalization, microbiological cultures, other imaging modalities, biopsies of pathological tissues, the empirical judgment of the clinician, and at least 3 months of clinical follow-up. The diagnostic efficacy of 18F-FDG PET/CT is assessed according to these multifaceted criteria.

When abnormal 18F-FDG uptake in organs or tissues correlates with clinical, imaging, and histopathological findings confirming it as the cause of the fever, it is classified as a “True Positive” (TP). Conversely, if the uptake is deemed unrelated to the fever’s cause or if the cause remains unidentified during follow-up, it is categorized as a “False Positive” (FP).

A “True Negative” (TN) classification is assigned when there is no abnormal 18F-FDG uptake, and one of the following conditions is met: the cause of the fever remains undetected after at least 3 months of clinical follow-up, the fever resolves spontaneously without specific treatment, or the patient succumbs to another illness unrelated to the fever.

Conversely, a “False Negative” (FN) is recorded when an infection, malignancy, or other disease is identified as the fever’s cause within the three-month follow-up, the fever persists beyond the follow-up period, or if the patient dies from FUO without a definitive diagnosis.

### 2.5 Data analysis

The data collected for this study were organized, tabulated, and analyzed using SPSS 27.0 (Statistical Product and Service Solutions) software. The diagnostic performance of 18F-FDG PET/CT for identifying active disease—including sensitivity, specificity, accuracy, positive predictive value (PPV), and negative predictive value (NPV)—was calculated based on standard definitions. Count data were represented as [*n* (%)]; Receiver operating characteristic (ROC) curve analysis was conducted using Medcalc software, with subsequent plotting of ROC curves. The DeLong test evaluated differences in the AUC between the new and existing models.

Additionally, factors such as age, gender, medical history, duration of fever, and various laboratory test results were analyzed as independent variables, with 18F-FDG PET/CT outcomes as the dependent variables. These outcomes were classified into two categories: true positive and non-true positive (encompassing false positive, false negative, or true negative). Odds ratios (OR) and 95% CI were computed for these classifications. A *p*-value of less than 0.05 was considered to indicate statistical significance.

Variables exhibiting a *p*-value of 0.10 or less in univariate analysis were further included in stepwise multivariate logistic regression models to refine the predictive accuracy. The significance level for tests was set at *α* = 0.05, where *p* < 0.05 indicated a statistically significant difference.

## 3 Results

### 3.1 Patient characteristics and final diagnosis

This study included 284 subjects, comprising 149 males (52.5%) and 135 females (47.5%). Fifteen patients (5.3%) had positive blood cultures, and four patients (1.4%) succumbed to their conditions. Most of 208 out of 284 patients (73.2%) received empirical antibiotic treatment, while 34 patients (12.0%) were treated with glucocorticoids before undergoing 18F-FDG PET/CT. Subsequent adjustments to the treatment regimen were made for 125 patients (44.0%) based on the PET/CT results. Age distribution was represented by a quartile range of 63 years (interquartile range: 47.25 to 69 years), hospitalization duration was 12 days (interquartile range: 8 to 16), duration of intermittent fever prior to admission was 25 days (interquartile range: 18.25, 40), and the peak temperature during fever episodes was 39°C (interquartile range: 38.7 to 39.5°C). The main demographic and clinical characteristics of the patients included in this study are summarized in Table 1.

**Table 1:** Basic information about the research subjects.

Characteristic	Categorization	[*n* (%), *M* (*P*_25_, *P*_75_)]
Genders	Male	149 (52.5)
	Female	135 (47.5)
Final diagnosis	Infectious diseases	53 (18.7)
	Malignant tumors	66 (23.2)
	Non-infectious inflammatory diseases	76 (26.8)
	FUO	89 (31.3)
Blood culture	Negative	269 (94.7)
	Positive	15 (5.3)
Vest	Existence	280 (98.6)
	Dead	4 (1.4)
Treatment change	No	159 (56.0)
	Yes	125 (44.0)
Age		63 (47.25, 69)
Days of hospitalization		12 (8, 16)
Number of days of intermittent fever before hospitalization		25 (18.25, 40)
High temperature at the time of fever		39 (38.7, 39.5)

Among the 284 cases enrolled, 53 patients (18.7%) were diagnosed with infectious diseases, including tuberculosis (TB, *n* = 3), Epstein–Barr virus and cytomegalovirus infection (*n* = 7), other viral infections (*n* = 4), brucellosis (*n* = 3), liver abscess (*n* = 5), abdominal infections (*n* = 1), pelvic infections (*n* = 1), parasitic infections (*n* = 2), infective pericarditis (*n* = 1), infective endocarditis (*n* = 1), sepsis (*n* = 10), pulmonary invasive aspergillosis (*n* = 1), co-infection with pseudoaneurysm of the head and arm trunks (*n* = 1), bronchiectasis with pneumonia (*n* = 11), and other unspecified infections (*n* = 2). Additionally, 76 patients (26.8%) were diagnosed with non-infectious inflammatory diseases, including vasculitis (*n* = 18), adult-onset Still’s disease (AOSD, *n* = 19), hemophagocytic syndrome (*n* = 7), necrotizing lymphadenitis (*n* = 14), IgG4-related disease (*n* = 2), eosinophilic dermatitis (*n* = 1), recurrent polychondritis (*n* = 2), arthritis (*n* = 5), interstitial pneumonia (*n* = 1), cutaneous lymphadenitis (*n* = 1), reactive lymphadenitis (*n* = 1), and undifferentiated connective tissue disease (UCTD) (*n* = 5). Furthermore, 66 patients (23.2%) were diagnosed with malignant tumors, including bladder cancer (*n* = 4), nasopharyngeal carcinoma (*n* = 1), lymphoma (*n* = 28), other hematologic malignancies (*n* = 1), lung cancer (*n* = 17), hepatocellular carcinoma (*n* = 3), renal cancer (*n* = 2), prostate cancer (*n* = 2), colorectal cancer (*n* = 3), duodenal adenocarcinoma (*n* = 1), adrenal carcinoma (*n* = 1), endometrial carcinoma (*n* = 1), squamous carcinoma of the groin (*n* = 1), and invasive squamous cell carcinoma of the skin (*n* = 1).

Ultimately, the cause of fever remained unknown in 89 patients (89/284, 31.3%), as detailed in Table 2.

**Table 2:** Disease spectrum profile of the study population.

Final diagnosis	Total cases	Number of true-positive PET/CT results
Infectious diseases	53	29
Virus infection	11	5
Bacterial infection	36	19
*Mycobacterium tuberculosis* infection	3	2
Parasitic and fungal infections	3	3
Malignant tumors	66	61
Solid tumor	37	34
Lymphomas	28	26
Other hematologic tumors	1	1
Non-infectious inflammatory diseases	76	46
Adult-onset Still’s disease	19	9
Vasculitis	18	17
Osteoarthritis	5	5
Necrotizing lymphadenitis	14	9
Other types of connective tissue diseases	20	6
Fever of unknown origin	89	0

### 3.2 Diagnostic performance of 18F-FDG PET/CT imaging

There is a debate regarding the diagnostic utility of 18F-FDG PET/CT for patients with FUO. Some scholars, such as Jaruskova and Belohlavek (12), assert that negative 18F-FDG PET/CT scans do not aid in diagnosing FUO, similar to the findings of Georgia et al. (13), who noted that scans other than true positives do not contribute to the diagnosis (4). Conversely, Keidar et al. (13) argue that true negatives can be crucial by essentially excluding focal infections, malignant tumors, arthritis, vasculitis, and other immune system disorders, which are vital for shaping the patient’s future treatment plan and improving prognosis.

In our study, only true-positive scans were considered beneficial for clinical diagnosis, as negative 18F-FDG PET/CT scans failed to elucidate the cause of the fever until the patient’s symptoms resolved spontaneously. A retrospective evaluation of the diagnostic performance of 18F-FDG PET/CT, based on the final clinical diagnosis of enrolled patients18F-FDG PET/CT imaging, identified characteristic changes corresponding to a confirmed diagnosis in 136 of the 284 patients with FUO (47.9%). 18F-FDG PET/CT may have contributed to the final clinical diagnosis in 47.9% of FUO cases. The sensitivity of 18F-FDG PET/CT in diagnosing FUO was 79.5% (136/171), the specificity was 61.1% (69/113), the positive predictive value was 75.6% (136/180), and the negative predictive value was 66.3% (69/104). Detailed sensitivity, specificity, and accuracy for each disease category are provided in Table 3.

**Table 3:** Diagnostic performance of 18F-FDG PET/CT in various disease categories.

Final diagnosis	Sensitivity	Specificity	Accuracy
Infectious diseases	54.7%	89.6%	83.1%
Malignant tumors	92.4%	91.3%	91.5%
Non-infectious inflammatory diseases	60.5%	96.2%	86.6%

### 3.3 Clinicopathologic features and 18F-FDG PET/CT findings

18F-FDG PET/CT is not recommended for general screening in the early stages of clinical assessment due to its high cost compared to other diagnostic tests. Therefore, timing the use of 18F-FDG PET/CT is crucial to maximize its diagnostic value for fever of FUO. This study collected clinicopathologic data including gender, age, medical history, white cell count, neutrophil percentage, lymphocyte percentage, C-reactive protein (CRP) levels, procalcitonin (PCT) levels, erythrocyte sedimentation rate (ESR), and ferritin levels as independent variables,18F-FDG PET/CT outcomes were analyzed as dependent variables through both univariate and multivariate logistic regression to determine the association of these risk factors with true-positive 18F-FDG PET/CT results. Only the most recent data preceding the 18F-FDG PET/CT exam were considered for statistical analysis. Our findings indicate that a clinical presentation of fever with localized pain and prolonged activated partial thromboplastin time (APTT) is associated with true-positive 18F-FDG PET/CT results, as detailed in Table 4 (14).

**Table 4:** Regression analysis of clinical history data and true-positive 18F-FDG PET/CT.

	Single factor regression analysis		Multifactor regression analysis	
Indicators	OR(95%)	*p*	OR(95%)	*p*
Chills	1.335 (0.83, 2.147)	0.233		
Muscle pain	0.62 (0.362, 1.064)	0.083		
Joint pain	0.909 (0.518, 1.593)	0.738		
Localized pain	2.108 (1.248, 3.559)	0.005	2.491 (1.398, 4.436)	0.002
Diabetes	1.529 (0.8, 2.924)	0.199		
High blood pressure	1.167 (0.702, 1.94)	0.552		
Chronic kidney disease	2.25 (0.662, 7.649)	0.194		
Chronic lung disease	0.589 (0.193, 1.805)	0.355		
Heart attack	0.872 (0.403, 1.887)	0.728		
Gout	1.647 (0.271, 10.007)	0.588		
Connective tissue disease	0.212 (0.024, 1.837)	0.159		
Disease of the blood	1.465 (0.322, 6.666)	0.622		
Surgical history	1.097 (0.666, 1.806)	0.717		
Allergy history	1.231 (0.647, 2.343)	0.527		
Previous antibiotic use	0.891 (0.527, 1.507)	0.667		
Prior glucocorticoid use	0.639 (0.307, 1.333)	0.233		
Hepatosplenomegaly	0.673 (0.37, 1.223)	0.194		
Lymph node Enlargement	1.168 (0.705, 1.935)	0.547		
Skin rash	0.831 (0.485, 1.422)	0.499		
Days of hospitalization	1.011 (0.983, 1.04)	0.429		
Age	1.003 (0.99, 1.017)	0.642		
Number of days of intermittent fever before hospitalization	1.002 (0.999, 1.005)	0.126		
High temperature at the time of fever	0.997 (0.692, 1.436)	0.986		
WBC	0.993 (0.957, 1.031)	0.729		
N%	1.252 (0.261, 6.004)	0.779		
L%	0.451 (0.086, 2.362)	0.346		
Creatinine	1.008 (1, 1.016)	0.053		
ALT	1.001 (0.999, 1.003)	0.442		
AST	0.999 (0.997, 1.002)	0.59		
Ferrous protein	1 (1, 1)	0.113		
Lactate dehydrogenase	1 (0.999, 1)	0.414		
Cholesterol	1.275 (0.983, 1.652)	0.067		
Triglyceride	0.876 (0.647, 1.188)	0.395		
PCT	1.005 (0.968, 1.044)	0.782		
CRP	1 (0.997, 1.003)	0.913		
ESR	1.004 (0.997, 1.011)	0.315		
APTT	0.939 (0.886, 0.996)	0.035	0.933 (0.876, 0.994)	0.031
PT	0.997 (0.88, 1.13)	0.964		
PTA	1.006 (0.991, 1.021)	0.443		
FBG	0.957 (0.851, 1.075)	0.455		
HB	1.003 (0.992, 1.013)	0.607		
PLT	0.999 (0.998, 1.001)	0.499		
Albumin	1.003 (0.962, 1.046)	0.889		

### 3.4 Cost-effectiveness of 18F-FDG PET/CT in diagnosing FUO

For patients with FUO 18F-FDG PET/CT, this imaging technique shows potential as a routine, cost-effective option. It can avoid unnecessary, invasive, and costly investigations while providing timely diagnostic clues that expedite the diagnostic process and reduce hospitalization duration. Although our study lacks a control group—since all participants underwent 18F-FDG PET/CT post-admission—previous research reveals its cost-effectiveness when performed early during hospitalization. A 2020 retrospective study revealed that patients undergoing 18F -FDG PET/CT within the first 7 days of admission experienced significantly fewer mean hospitalization days and lower healthcare costs before diagnosis than those scanned after 7 days (15). Another study compared 46 FUO patients who underwent 18F-FDG PET/CT upon admission against 46 who did not, finding that the latter group had more extended hospital stays (21 days vs. 6.9 days) and higher costs (€5,298 vs. €12,614) (16).

The early use of 18F -FDG PET/CT in the diagnostic workflow can facilitate prompt diagnosis, streamline decision-making, shorten hospital stays, and reduce costs by avoiding redundant tests. Additionally, some researchers highlight that early 18F-FDG PET/CT applications can allow for timely adjustments in treatment regimens, enhancing therapeutic outcomes. For instance, a retrospective study (17) observed that 53% of FUO patients had their treatment regimens adjusted 18F-FDG PET/CT. In our study, 44% (125/284) of patients had adjustments made to their treatment 18F-FDG PET/CT. However, due to the retrospective nature of these studies, it is sometimes challenging to directly attribute clinical treatment adjustments to 18F-FDG PET/CT results, particularly when changes involve the same class of drugs. For example, most patients suspected of having connective tissue disease in our study were already receiving empirical treatment with glucocorticoids, immunosuppressants, or nonsteroidal anti-inflammatory drugs before undergoing 18F-FDG PET/CT, and some reported symptomatic relief, leading to minimal changes in their treatment approach post-imaging.

## 4 Discussion

In their seminal study defining FUO, Petersdorf and Beeson identified the etiologies as infectious diseases (36%), tumors (19%), and miscellaneous or unknown causes (26%) (1). Since that time, the disease characteristics of FUO have evolved significantly due to advancements in diagnostic technologies. A recent cohort study by Yong et al. (18). analyzed retrospective data from 2002 to 2012, revealing that in northern China, the prevalence of infectious diseases, NIID, malignant tumors, and other undiagnosed cases was 51.5, 18.4, 11.9, and 7.1%, respectively. Their analysis of six studies from Western countries between 2003 and 2016 showed frequencies of 29.1% for infectious diseases, 25.8% for NIID, 11.6% for malignant tumors, and 26.1% for other undiagnosed cases.

Currently, infectious diseases are the predominant cause of FUO. Another study retrospectively analyzed data from 2005 to 2015, encompassing 18 studies and 3,164 patients, and identified infectious diseases as the primary cause (19). Most participants in this study were from Jilin Province in northern China, which features a subtropical, inland, and dry climate. The incidence of infectious diseases in this study (18.7%, 53/284) was lower compared to the broader region of northern China. However, the incidences of NIID (26.8%, 76/284) and malignant tumors (23.2%, 66/284) were higher. These discrepancies likely stem from regional environmental, geographic, and economic differences. For instance, healthcare teams in infectious disease departments may conduct pathogen identification tests more rapidly, leading to earlier detection of infectious diseases before cases are classified as FUO. Furthermore, the preliminary use of antibiotic therapy prior to examinations with ^18F-FDG PET/CT might influence the diagnosis of infectious diseases.

These variations are also attributable to advanced diagnostic techniques and the continuous refinement of diagnostic approaches. For example, the rising frequency of diagnosed malignant tumors may relate to enhanced cancer detection methods in hospitals. Fusco et al. (19) observed a significant increase in confirmed NIID diagnoses over time, which they attributed not only to advances in medical knowledge and greater awareness among clinicians but also to the integration of basic immunologic tests into the FUO diagnostic process, thereby aiding in the identification of NIID clues in patients. Therefore, optimizing FUO diagnostic strategies should consider the prevalent causes of fever in different regions, local epidemiology, and available resources.

Minamimoto et al. (20) synthesized findings from previous meta-analyses on the diagnostic role of 18F-FDG PET/CT in managing FUO, demonstrating that this imaging technique offers high sensitivity (79.5%, 136/171) and moderate specificity (61.1%, 69/113) for FUO diagnosis. These figures align with the results from two recent meta-analyses that also support the utility of 18F-FDG PET/CT in this context (21–23).

However, the absence of a universally accepted gold standard for FUO complicates comparisons of sensitivity and specificity across different studies due to variations in patient characteristics and diagnostic test sequencing. Therefore, assessing the clinical usefulness of 18F-FDG PET/CT, rather than its sensitivity and specificity alone, becomes more relevant (7). Kouijzer et al. (3) concluded that 18F-FDG PET/CT is particularly valuable when it leads to a definitive etiological diagnosis of FUO, contributing to the final diagnosis in 38 to 75% of cases. In this specific study, 18F-FDG PET/CT proved helpful in 47.9% (136/284) of cases and contributed to the final diagnosis in 69.7% (136/195) of the 195 patients who received a definitive diagnosis, consistent with earlier research (7, 24, 25).

The timing of 18F-FDG PET/CT in the diagnostic process is critical; earlier use can potentially alter the course of the investigation, as preliminary blood results and other imaging studies might already provide diagnostic clues, discouraging further testing and reserving ^18F-FDG PET/CT for more challenging cases.

Moreover, the pre-examination use of empirical treatments, such as glucocorticoids or antibiotics, remains contentious. In this cohort, 73.2% (208/284) of patients were given antibiotics, and 12.0% (34/284) received glucocorticoids before undergoing 18F-FDG PET/CT. While some studies suggest that antibiotics might reduce the detection of infected lesions in 18F-FDG PET/CT (26), others argue that antibiotics do not significantly impact diagnostic accuracy. Conversely, corticosteroid use is associated with false-negative results (27), particularly affecting the detection of inflammatory diseases such as vasculitis and polymyalgia rheumatica. The prolonged use of corticosteroids before 18F-FDG PET/CT examinations tends to have a more significant negative impact, thus halting their use prior to imaging is recommended to enhance diagnostic accuracy (28, 29).

In this study, 89 patients remained with an unresolved cause of fever; 69 of these individuals exhibited no positive results from 18F-FDG PET/CT scans. Among these, one patient died due to diabetic complications, one from a cerebral infarction, and two from cardiac infarctions. The remaining 65 patients saw their fever symptoms resolve post-discharge, as confirmed through subsequent follow-up. Retrospective analysis suggests a high likelihood of spontaneous fever resolution in patients without anemia or hypoalbuminemia (30). Takeuchi et al. (31) similarly noted that patients with FUO, who lacked a definitive diagnosis after extensive diagnostic evaluations and had negative 18F-FDG PET/CT results, were likely to experience spontaneous symptom remission.

Kouijzer et al. (3) observed that in cases of persistent FUO with negative 18F-FDG PET/CT results, it may be more prudent to await the emergence of new diagnostic clues rather than to proceed immediately with further testing. Therefore, if 18F-FDG PET/CT does not yield significant positive findings, additional testing might not be recommended; instead, a wait-and-see approach or the empirical application of NSAIDs and corticosteroid therapy could be considered in the clinical management of patients with undiagnosed FUO (3, 32–34).

**Figure 1:**
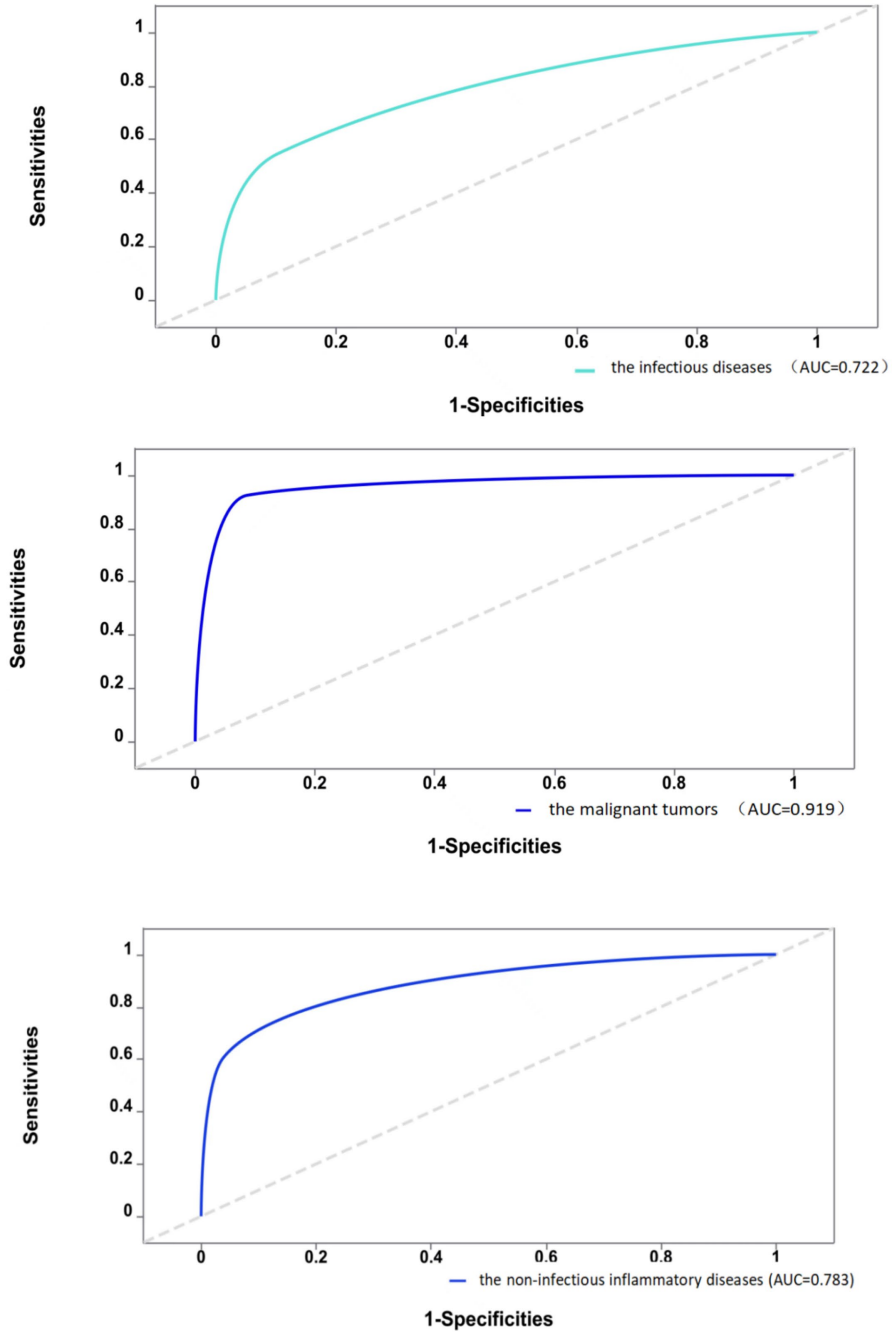
Diagnostic efficacy of 18F-FDG PET/CT in different types of diseases.

Furthermore, this study assessed the diagnostic efficacy of 18F-FDG PET/CT in identifying different types of diseases causing FUO through ROC analysis, as detailed in Figure 1. The ROC analysis showed an AUC of 0.722 for infectious diseases, with a sensitivity of 54.7% and a specificity of 89.6%. The analysis for non-infectious inflammatory diseases showed an AUC of 0.783, sensitivity of 60.5%, and specificity of 96.2%. The AUC was notably higher for malignant tumors at 0.919, with a sensitivity of 92.4% and a specificity of 91.3%. Thus, compared to infectious and non-infectious inflammatory diseases,18F-FDG PET/CT demonstrates greater sensitivity and specificity in diagnosing malignant diseases.

The final diagnosis of a disease relies on a multifactorial analysis rather than the outcome of a single diagnostic test. This study evaluated whether the diagnostic efficacy improved when 18F-FDG PET/CT results were integrated into the existing diagnostic model. The original model included five independent variables: CRP, ESR, PCT, leukocytes, and ferritin. The new model incorporated the results of 18F-FDG PET/CT alongside these existing variables.

To assess the effectiveness of the updated model, we employed the Delong test to analyze the difference in the AUC between the old and new models. A positive NRI indicates that the revised model offers superior diagnostic capabilities compared to its predecessor. The findings revealed that the AUC for the new model was superior in diagnosing infectious diseases, malignant neoplastic diseases, and non-infectious inflammatory diseases. This enhancement suggests that including 18F-FDG PET/CT results significantly improves predictive efficacy. As detailed in Table 5, integrating 18F-FDG PET/CT findings into the diagnostic process mainly benefits the management of suspected malignancies causing FUO, showing the heightened diagnostic performance of 18F-FDG PET/CT in these scenarios. This refined approach allows for more accurate and timely identification of the underlying causes of FUO, facilitating more targeted and effective clinical interventions (35).

**Table 5:** Delong test results between the new and the old models.

Type of disease	Old model		New model		Statistic	*p*	NRI
	AUC	95%CI	AUC	95%CI			
Infectious diseases	0.634	0.556–0.712	0.774	0.696–0.852	−3.751	0.0002	0.858
Malignant tumors	0.664	0.591–0.738	0.931	0.889–0.973	−7.3795	<0.001	1.647
Non-infectious inflammatory diseases	0.708	0.641–0.775	0.857	0.805–0.910	−4.993	<0.001	1.122

**Figure 2:**
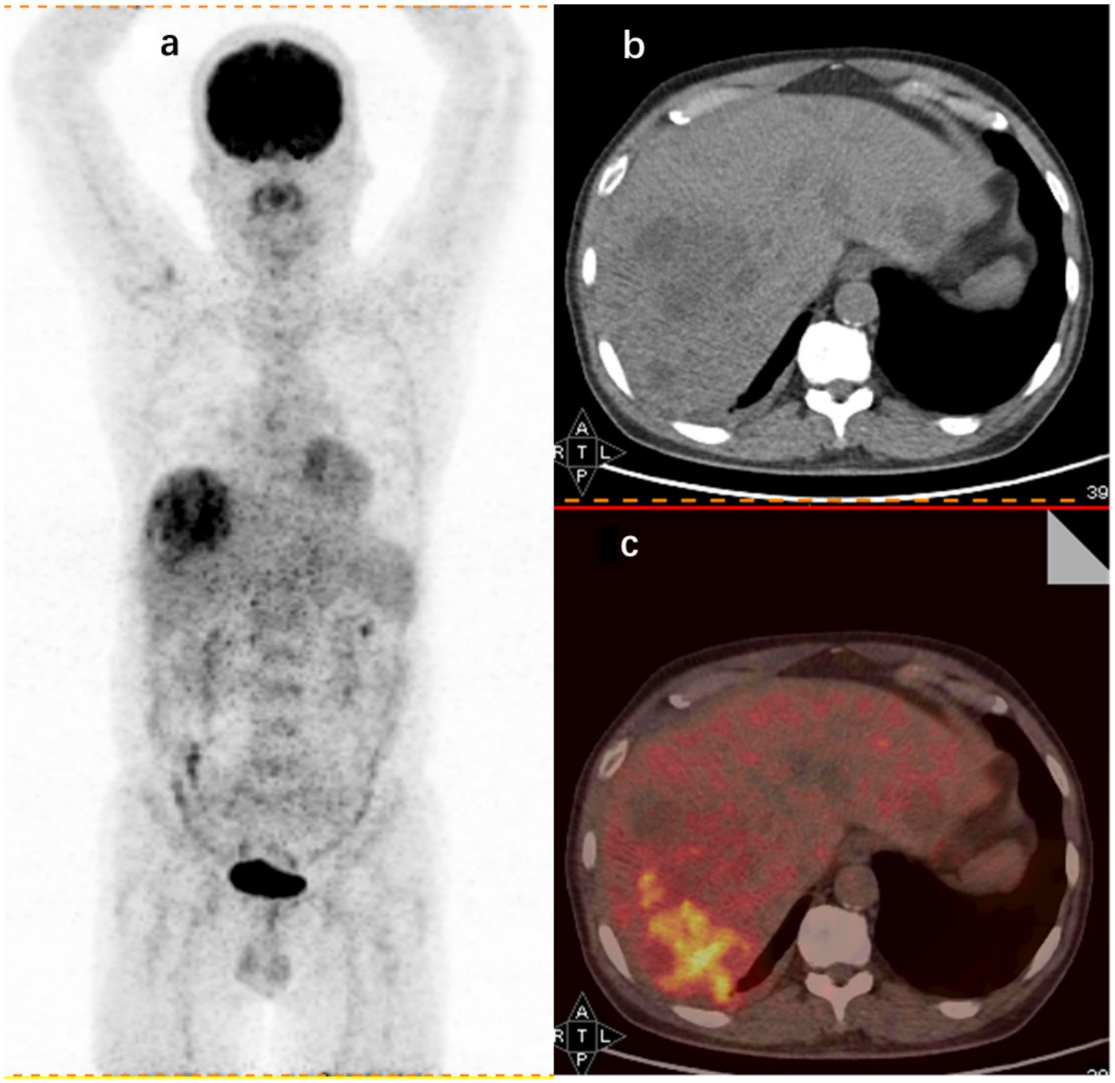
18F-FDG PET/CT findings in a 60-year-old male FUO patient with a final diagnosis of liver abscess. **(A)** Whole-body 18F-FDG PET coronal section showing an aggregated foci of metabolic activity in the right hepatic lobe. CT axial section **(B)** and imaging fusion **(C)** showing foci of increased uptake in the right hepatic lobe (SUVmax 8.1).

This study also explored the specific diagnostic capabilities of 18F-FDG PET/CT for different causes of FUO. Over the past decade, 18F-FDG PET/CT has become a staple in tumor imaging due to its ability to detect cells with high glycolytic activity. However, 18F-FDG is not specific to tumor cells; it also accumulates in activated leukocytes (36), which is pivotal in its application beyond oncology. Since the initial observation of high FDG uptake in abdominal abscesses (37), 18F-FDG PET/CT has been extensively utilized in diagnosing and managing infectious diseases. In the context of this study, abnormal 18F-FDG PET/CT findings were present in all patients diagnosed with liver abscesses. This is consistent with early findings that FDG is taken up by large numbers of neutrophils and lymphocytes at infection sites, which supports the idea (38) that 18F-FDG PET/CT is particularly effective in identifying abscessed lesions due to their significant leukocyte infiltration compared to other infections. For illustration, Figure 2 in this study depicts a hypermetabolic lesion in the right hepatic lobe of a 60-year-old patient with an FUO-related liver abscess.

18F-FDG PET/CT also demonstrates considerable diagnostic value for identifying lymphoma as a cause of fever of FUO. This study identified 28 of the 66 patients diagnosed with malignant tumors with lymphoma. Among these, 18F-FDG PET/CT imaging strongly suggested lymphoma in 26 patients, aligning with their final diagnoses. However, two patients presented with 18F-FDG PET/CT findings indicative of inflammatory lesions but were later diagnosed with lymphoma, highlighting some challenges in differential diagnosis.

Conversely, there were 17 cases where 18F-FDG PET/CT was highly suggestive of lymphoma, yet subsequent pathologic evaluation of lymph node biopsies ruled out lymphoma. These patients were diagnosed with other conditions: three with necrotizing lymphadenitis, 2 with Adult Still’s disease, 2 with mixed hepatocellular carcinoma-cholangiocarcinoma, 2 with hemophagocytic syndrome, and 1 with an infectious disease. The remaining seven cases remained undiagnosed, although their conditions improved following relevant treatments.

The sensitivity of 18F-FDG PET/CT in diagnosing lymphoma in patients with FUO was notably high at 92.9% (26/28) with a PPV of 60.5% (26/43). Another study supports these findings and emphasizes the importance of 18F-FDG PET/CT in the diagnostic workflow for suspected lymphoma in FUO cases. Additionally, the study developed a lymphoma prediction model based on 18F-FDG PET/CT characteristics, further enhancing the diagnostic efficiency for FUO (39).

Retrospective analyses have explored the prognostic capabilities of 18F-FDG PET/CT parameters such as maximum standardized uptake value (SUVmax), whole-body metabolic tumor volume, and whole-body total lesion glycolysis (40, 41). These metrics are instrumental in not only diagnosing but also predicting the prognosis of lymphoma in FUO patients.

**Figure 3:**
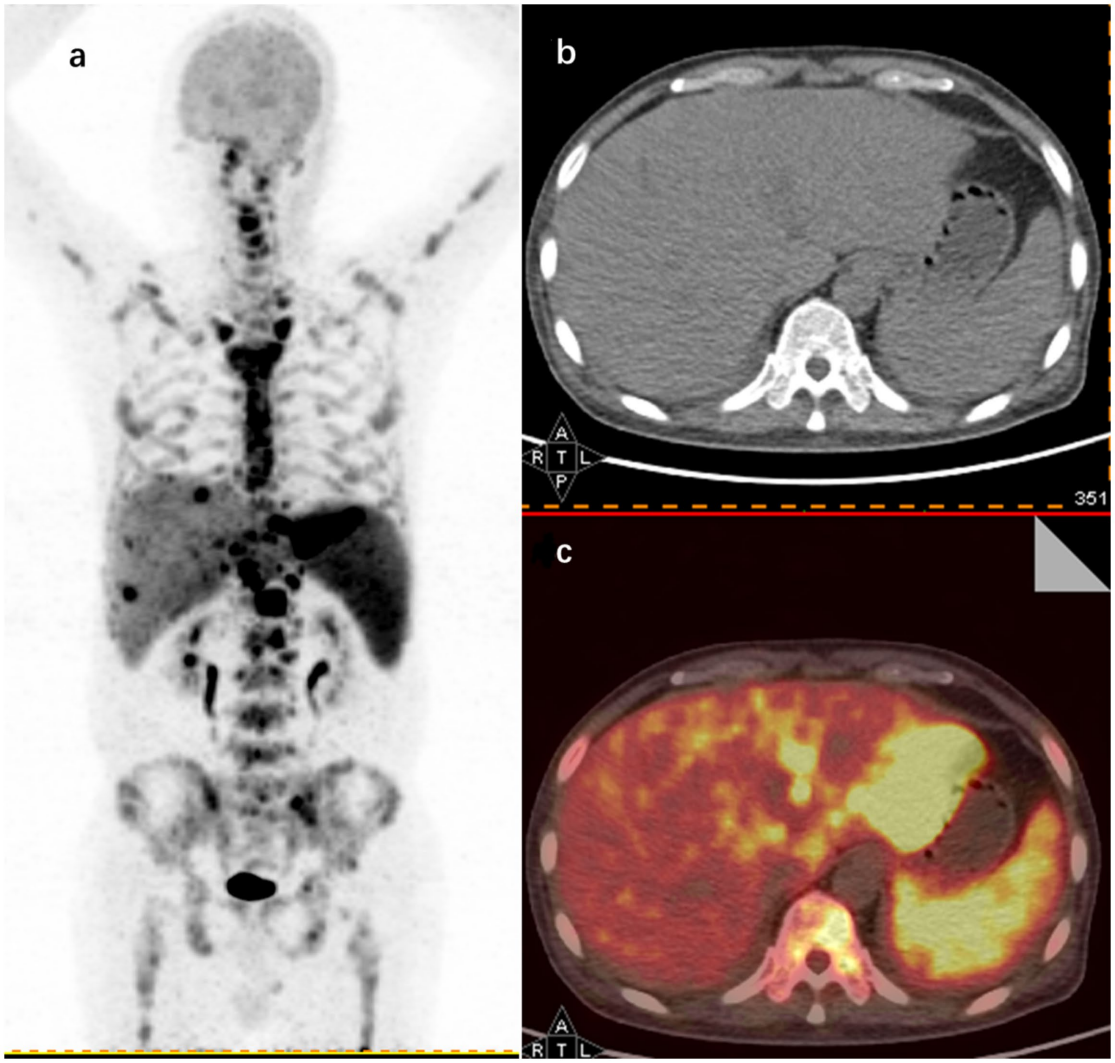
18F-FDG PET/CT findings in a 62-year-old male FUO patient with a final diagnosis of lymphoma. **(A)** Maximum intensity projection (MIP) images of whole-body 18F-FDG PET coronal sections show hypermetabolic sites in the left hepatic lobe (SUVmax 23.1), spleen (SUVmax 8.5), and throughout the skeleton (SUVmax 16.8). Representative axial sections of CT **(B)** and image fusion **(C)** show increased uptake in the left hepatic lobe and spleen.

Illustratively, Figure 3 in this study shows 18F-FDG PET/CT detecting hypermetabolic activity in the left liver lobe, spleen, and bones of a 62-year-old patient with FUO, who was later diagnosed with lymphoma.

In this study, NIID emerged as the second most frequent cause of FUO (accounting for 26.8%, 76/284) cases, with vasculitis being particularly prevalent within this group, representing 23.7% (18/76) of the NIID cases. 18F-FDG PET/CT played a pivotal role in diagnosing these conditions, aligning with the final diagnosis of vasculitis in 17 of the 18 patients diagnosed with this condition. The high diagnostic yield of 18F-FDG PET/CT for detecting active large vessel vasculitis is well-documented (42–44). The utility of 18F-FDG PET/CT in this context is indicated by its favorable diagnostic performance, making it a valuable tool in the clinical evaluation of patients with suspected active large vessel vasculitis.

**Figure 4:**
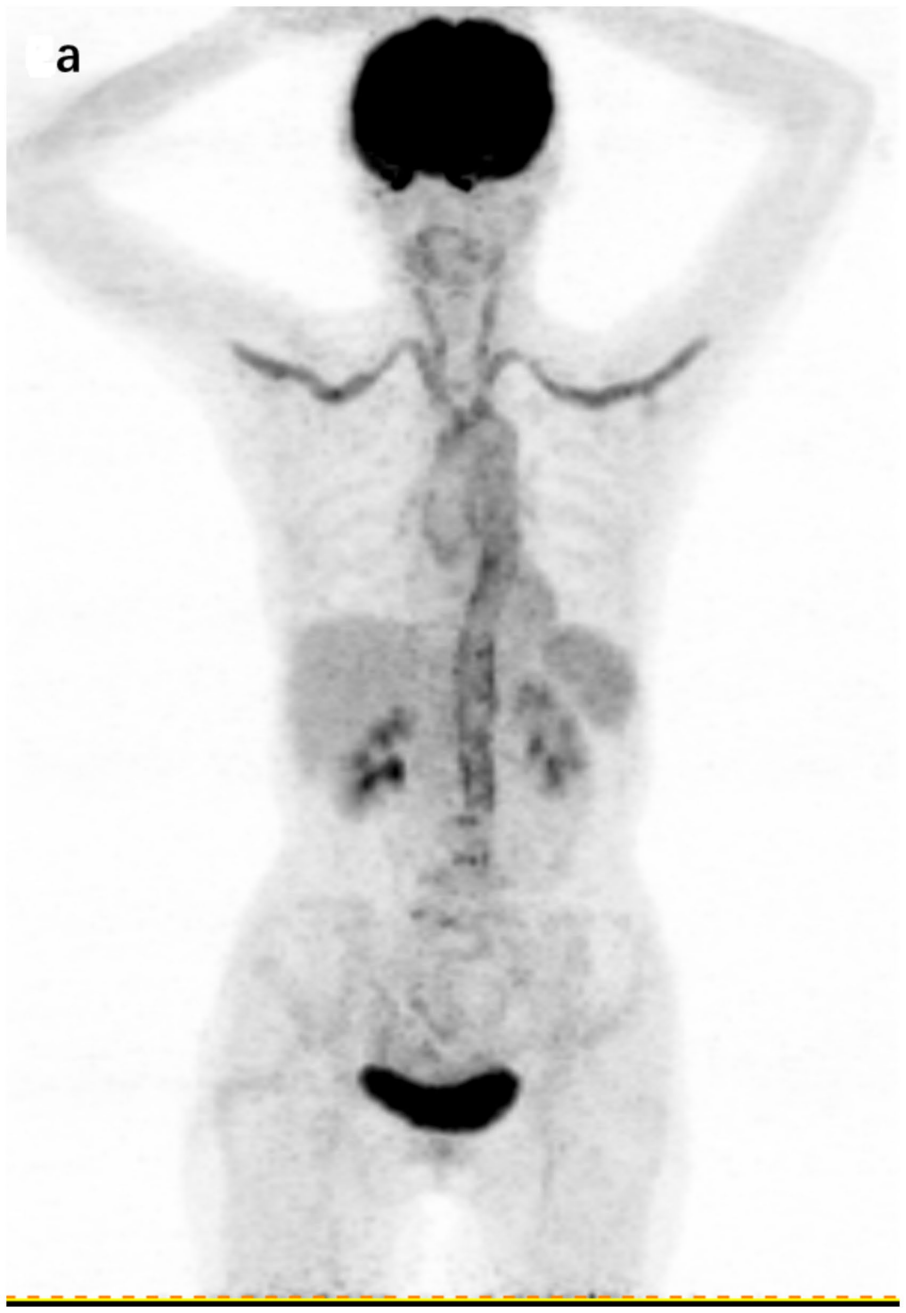
18F-FDG PET/CT findings in a 72-year-old patient with a final diagnosis of large vessel vasculitis. **(A)** Coronal section of whole-body 18F-FDG PET showing cumulative FDG uptake in the aorta, subclavian artery, and carotid artery (SUVmax 5.2).

Illustratively, Figure 4 highlights the capabilities of 18F-FDG PET/CT in this regard. The imaging detected a significant accumulation of FDG, with a maximum SUV of 5.2, in the aorta, subclavian arteries, and carotid arteries of a 72-year-old patient diagnosed with large vessel vasculitis.

In recent years, researchers have started leveraging traditional risk factors to enhance the diagnostic performance of 18F-FDG PET/CT for FUO by identifying characteristics of patients who are most likely to benefit from this imaging modality. Our study observed a notable correlation between localized pain, prolonged APTT, and true-positive 18F-FDG PET/CT results.

Prolonged APTT can occur in various febrile conditions, though the mechanisms behind this are not fully understood. For instance, one study linked prolonged APTT in severe fever with thrombocytopenia syndrome (SFTS) to a deficiency in coagulation factor XI caused by SFTS virus infection (45). Similarly, another study indicated that in dengue hemorrhagic fever, prolonged APTT may result from the secretion of dengue virus nonstructural protein 1 (NS 1), which inhibits plasminogen activation (46). These complex underlying mechanisms warrant further investigation.

Moreover, other variables have been identified as relevant in previous studies. Mahajna et al. (11) found associations between weight loss, low hemoglobin levels, and diagnostic outcomes via 18F-FDG PET/CT. Another retrospective study suggested that combining 18F-FDG PET/CT findings with CRP levels could improve the accuracy of FUO diagnoses (47). A comprehensive retrospective analysis involving 498 patients established the predictive value of CRP and ESR in producing positive 18F-FDG PET/CT results. Notably, elevated CRP levels more accurately reflected the presence and extent of inflammation compared to ESR, with the study reporting a 100% true negative rate for 18F-FDG PET/CT in patients with CRP levels below 5 mg/L. However, while diagnosis rates increased with rising CRP levels, no optimal CRP threshold was determined (14).

Crouzet et al. (48) found significant correlations between high CRP levels (>30 mg/L), anemia, and beneficial 18F-FDG PET/CT results. Contrarily, Bleeker-Rovers et al. (49) noted that 18F-FDG PET/CT may not be indicated for FUO patients with normal CRP and ESR levels. These differences among studies can be attributed to variations in research methodology, including how true positives are defined (i.e., whether they are useful solely for diagnosis or include both true positives and true negatives).

Additionally, while CRP and white blood cell counts generally indicate infection and inflammation, their patterns may not uniformly align across different diseases. For example, high CRP levels are common in autoinflammatory diseases, such as vasculitis and arthritis, even when white blood cell counts remain normal or only moderately elevated (17).

To address this, our study also examined the predictive value of the same laboratory indicators for true-positive 18F-FDGPET/CT results across different disease types. Surprisingly, only patients with a history of chronic kidney disease who were diagnosed with non-infectious inflammatory diseases demonstrated a higher likelihood of a true-positive 18F-FDG PET/CT result, a finding not previously reported. This novel insight is detailed in Tables 6–8.

**Table 6:** Infectious diseases.

Indicators	Single-factor regression analysis		Multifactor regression analysis	
	OR (95%)	*p*	OR (95%)	*p*
Chills	1.31 (0.44–3.89)	0.631		
Muscle pain	0.16 (0.04–0.68)	0.013	0.24 (0.03–2.30)	0.218
Joint pain	0.80 (0.18–3.61)	0.771		
Localized pain	0.45 (0.14–1.44)	0.178		
Chronic kidney disease	0.18 (0.02–1.72)	0.136		
Chronic lung disease	0.11 (0.01–0.97)	0.046	2.78 (0.08–97.44)	0.573
Heart attack	0.25 (0.07–0.86)	0.029	0.21 (0.02–2.17)	0.191
Gout	0.25 (0.02–2.58)	0.244		
Connective tissue disease	0.10 (0.00–3.38)	0.199		
Disease of the blood	0.09 (0.00–2.03)	0.132		
Hepatosplenomegaly	1.04 (0.25–4.41)	0.956		
Lymph node Enlargement	0.03 (0.01–1.14)	<0.001	0.16 (0.02–1.16)	0.070
Skin rash	0.81 (0.15–4.42)	0.806		
WBC	0.83 (0.72–0.96)	0.013	1.06 (0.83–1.37)	0.626
*N*%	2.62 (0.06–116.72)	0.619		
L%	0.11 (0.00–8.65)	0.319		
HB	1.01 (0.98–1.04)	0.409		
PLT	1.00 (0.99–1.00)	0.100		
Creatinine	0.98 (0.97–1.00)	0.087		
ALT	1.00 (1.00–1.00)	0.639		
AST	1.00 (0.99–1.00)	0.618		
Albumin	0.97 (0.88–1.08)	0.585		
Ferrous protein	1.00 (1.00–1.00)	0.452		
Lactate dehydrogenase	1.00 (1.00–1.00)	0.356		
Cholesterol	0.88 (0.50–1.55)	0.662		
Triglyceride	0.52 (0.23–1.13)	0.098		
PCT	1.08 (0.89–1.30)	0.454		
CRP	1.00 (0.99–1.00)	0.400		
ESR	0.99 (0.98–1.01)	0.402		
APTT	0.99 (0.88–1.12)	0.908		
PT	1.11 (0.85–1.44)	0.448		
PTA	0.99 (0.96–1.03)	0.642		
FBG	0.80 (0.59–1.08)	0.143		

**Table 7:** Non-infectious inflammatory diseases.

Indicators	Single-factor regression analysis		Multifactor regression analysis	
	OR (95%)	*p*	OR (95%)	*p*
Chills	0.42 (0.16–1.09)	0.075		
Muscle pain	2.34 (0.80–6.88)	0.121		
Joint pain	0.67 (0.26–1.71)	0.401		
Localized pain	0.31 (0.12–0.82)	0.018	0.23 (0.02–2.84)	0.251
Chronic kidney disease	0.04 (0.00–0.32)	0.003	0.03 (0.00–0.92)	0.044
Chronic lung disease	2.44 (0.03–212.56)	0.696		
Heart attack	0.35 (0.08–1.59)	0.173		
Gout	1.36 (0.00–597.98)	0.921		
Connective tissue disease	1.37 (0.02–103.81)	0.888		
Disease of the blood	1.38 (0.06–30.67)	0.838		
Hepatosplenomegaly	0.62 (0.14–2.69)	0.522		
Lymph node Enlargement	0.26 (0.09–0.70)	0.008	0.10 (0.01–1.19)	0.069
Skin rash	2.13 (0.72–6.29)	0.169		
WBC	0.98 (0.91–1.06)	0.567		
*N*%	0.09 (0.00–3.16)	0.186		
L%	10.79 (0.24–494.01)	0.223		
HB	1.01 (0.98–1.03)	0.583		
PLT	1.01 (1.00–1.01)	0.006	1.00 (0.99–1.01)	0.453
Creatinine	1.00 (0.98–1.03)	0.690		
ALT	1.00 (0.99–1.01)	0.917		
AST	1.00 (0.99–1.00)	0.504		
Albumin	1.01 (0.92–1.10)	0.896		
Ferrous protein	1.00 (1.00–1.00)	0.777		
Lactate dehydrogenase	1.00 (1.00–1.00)	0.440		
Cholesterol	1.31 (0.75–2.31)	0.346		
Triglyceride	0.82 (0.42–1.60)	0.569		
PCT	1.25 (0.76–2.05)	0.382		
CRP	1.00 (1.00–1.01)	0.549		
ESR	1.02 (1.00–1.03)	0.066		
APTT	0.97 (0.87–1.07)	0.519		
PT	1.22 (0.86–1.74)	0.264		
PTA	0.98 (0.94–1.01)	0.158		
FBG	1.59 (1.20–2.10)	0.001	0.79 (0.36–1.72)	0.548

**Table 8:** Malignant tumors.

Indicators	Single factor regression analysis		Multifactor regression analysis	
	OR (95%)	*p*	OR (95%)	*p*
Chills	0.60 (0.09–3.87)	0.595		
Muscle pain	0.29 (0.04–1.99)	0.210		
Joint pain	0.67 (0.26, 1.70)	0.401		
Localized pain	0.32 (0.05–2.10)	0.238		
Chronic kidney disease	0.08 (0.01–0.65)	0.019	0.14 (0.01–1.44)	0.099
Chronic lung disease	4.95 (0.01–2135.30)	0.605		
Heart attack	0.35 (0.08–1.58)	0.173		
Gout	0.09 (0.00–37.08)	0.428		
Connective tissue disease	0.09 (0.00–37.08)	0.428		
Disease of the blood	0.03 (0.00–7.49)	0.206		
Hepatosplenomegaly	1.81 (0.19–17.29)	0.607		
Lymph node Enlargement	0.35 (0.05–2.26)	0.270		
Skin rash	2.13 (0.72–6.28)	0.170		
WBC	0.99 (0.86–1.14)	0.867		
*N*%	0.09 (0.00–3.19)	0.189		
L%	0.07 (0.00–26.49)	0.382		
HB	1.01 (0.97–1.05)	0.772		
PLT	1.00 (0.99–1.01)	0.822		
Creatinine	1.00 (0.97–1.03)	0.830		
ALT	0.99 (0.98–1.00)	0.039	1.00 (0.99–1.01)	0.949
AST	0.99 (0.99–1.00)	0.103		
Albumin	1.14 (0.92–1.41)	0.228		
Ferrous protein	1.00 (1.00–1.00)	0.552		
Lactate dehydrogenase	1.00 (1.00–1.00)	0.093		
Cholesterol	1.76 (0.52–5.92)	0.361		
Triglyceride	13.03 (0.36–476.74)	0.162		
PCT	0.99 (0.91–1.08)	0.793		
CRP	1.00 (0.98–1.01)	0.618		
ESR	0.99 (0.97–1.02)	0.544		
APTT	1.00 (0.82–1.23)	0.977		
PT	1.00 (0.59–1.70)	0.999		
PTA	1.00 (0.95–1.06)	0.982		
FBG	0.87 (0.53–1.42)	0.571		

There are several limitations in our study. Firstly, the definition of FUO in some current studies is not uniform and lacks a diagnostic gold standard, which may introduce some errors in comparison. Secondly, due to the retrospective design of this study, the diagnostic workup of patients prior to 18F-FDG PET/CT was not fully standardized. Clinicians determined the patient’s overall course of treatment at their discretion, including adjusting treatment regimens and selecting examination sequences. Therefore, we could not ascertain whether comprehensive preliminary evaluations were conducted on every FUO patient before performing 18F-FDG PET/CT. Thirdly, there is controversy among various studies regarding whether true-negative 18F-FDG PET/CT results have diagnostic value, introducing some bias when comparing the diagnostic utility of 18F-FDG PET/CT across different studies. Fourthly, because this was a retrospective study, all patients enrolled underwent 18F-FDG PET/CT; thus, a control group was lacking.

## 5 Conclusion

The use of 18F-FDG PET/CT in our department has aided in the diagnosis of tuberculosis, abscesses, solid tumors, lymphomas, and vasculitis in patients with FUO and achieved high diagnostic accuracies in those diseases. Since early potential diagnostic clues can be obtained from 18F-FDG PET/CT, unnecessary further testing can be avoided, thus facilitating the initiation of the most effective treatments more rapidly and improving the overall prognosis of patients. The identifications of clinical factors that related to true-positive PET/CT diagnosis could further improve the diagnostic accuracy and facilitate more effective imaging allocation.

## Data Availability

The raw data supporting the conclusions of this article will be made available by the authors, without undue reservation.
